# Association analysis of the differences in intestinal flora and clinical tumor indicators among colorectal cancer patients

**DOI:** 10.3389/fcimb.2026.1742672

**Published:** 2026-02-23

**Authors:** Lijun Ma, Wenjing Wang, Shihu Ma, Yanbai Wang, Hai Li, Ying Gao, Xiaoliang Xie

**Affiliations:** 1 Department of Human Anatomy, Histology and Embryology, School of Basic Medicine, Ningxia Medical University, Yinchuan, China; 2 Department of Colorectal Surgery, General Hospital of Ningxia Medical University (The First Clinical Medical College of Ningxia Medical University), Yinchuan, China; 3 Department of Oncology, Ningxia Hui Autonomous Region People's Hospital, Yinchuan, China

**Keywords:** clinical tumor indicators, colorectal cancer, human, intestinal flora, precision medicine

## Abstract

**Background:**

Colorectal cancer (CRC) is the third most common malignant tumor globally, and its development is closely related to intestinal flora dysbiosis. However, the heterogeneity of cancerous tissues, paracancerous tissues, and fecal flora, and their clinical significance, has not been fully elucidated.

**Aim:**

This study aimed to systematically analyze the diversity, composition, and functional differences of intestinal flora in patients with CRC compared to healthy individuals, and to reveal potential associations between the characteristics of these microbial communities and tumorigenesis and development.

**Methods:**

Thirty CRC patients (30 cancerous tissue samples, 30 paracancerous tissue samples, and 30 fecal samples) and 30 healthy volunteers (30 fecal samples) were enrolled in the study. The microbial communities were analyzed using 16S rRNA sequencing, and the status of the bacterial flora was evaluated by combining alpha and beta diversity, species difference analysis, the Gut Microbiome Health Index (GMHI), and the Gut Microbiome Dysbiosis Index (MDI). The correlation of these factors with clinical parameters was then analyzed.

**Results:**

The alpha diversity of the cancerous tissue from patients with CRC was significantly lower than that of the fecal samples (*p* < 0.05). The intestinal microbiota of patients with CRC was statistically different from that of healthy individuals (*p* < 0.01). Additionally, there was a statistically significant difference in beta diversity between the cancerous tissue and fecal gut microbiota of patients with CRC (*p* < 0.01). The microbiota of the paracancerous tissues exhibited significantly higher GMHIs than the cancerous tissues. Healthy individuals demonstrated better gut health than individuals with CRC. The fecal samples from CRC patients had a higher GMHI than the cancerous tissues. The difference was statistically significant (*p* < 0.001). For MDI, however, the trend was reversed. A statistically significant positive correlation was observed between *Escherichia coli* and tumor size (*p* < 0.05). Similarly, *Methylobacterium/Methylorubrum* exhibited a statistically significant positive correlation with tumor stage (*p* < 0.05).The research found that Blautia and Faecalibacterium had higher abundances in the feces of healthy individuals and the tissues adjacent to colorectal cancer, while Escherichia-Shigella, Bacteroides, Enterococcus, and Fusobacterium had higher abundances in colorectal cancer tissues.

**Conclusion:**

The intestinal flora of CRC patients is characterized by decreased diversity, an enrichment of pathogenic bacteria, and a reduction in protective flora. these microbial alterations are associated with tumor progression, potentially via inflammatory and metabolic pathways, although causal mechanisms remain to be functionally validated. The flora health index and dysbiosis index have potential for use as adjunctive diagnostic tools. However, individualized preventive intervention strategies need to be developed in the future by combining multi-omics data.

## 1 Introduction

Colorectal cancer (CRC) is the third most common malignant tumor worldwide and one of the leading causes of cancer-related deaths (Bray et al., 2024). In recent years, the role of intestinal flora as the “second genome” in the occurrence, development, and treatment of CRC has received widespread attention. Studies have shown that dysregulation of the composition and function of intestinal flora (dysbiosis) is closely related to the progression of CRC. This dysregulation promotes tumorigenesis through metabolites (e.g., secondary bile acids, short-chain fatty acids (SCFAs)), immune regulation, inflammatory response, and DNA damage (González et al., 2024). For example, specific pathogenic bacteria such as *Fusobacterium nucleatum*, enterotoxin-producing *Escherichia coli*, and *Bacteroides* such as *Bacteroides fragilis* can directly contribute to the development of CRC by activating the Wnt/β-catenin signaling pathway or by producing genotoxins (Qu et al., 2023). In addition, the diversity and abundance of the intestinal flora are significantly reduced in CRC patients, and there is spatial heterogeneity in the flora characteristics of different anatomical sites (e.g., tumor tissue, paracancerous mucosa, normal mucosa, and feces) (Li et al., 2022). It has also been found that gut flora characteristics are associated with CRC recurrence and prognosis. A Chinese cohort study showed abnormal abundance of *Bacteroides* and *Prevotella* in the mucosal flora of patients with relapse, suggesting their potential as prognostic markers (Huo et al., 2022). However, the causal relationship between flora and CRC needs to be further verified. In addition, future studies need to integrate multi-omics data and develop individualized flora intervention protocols in combination with clinical characteristics.

Recent studies have revealed the unique role of intratumoral microbiota in the tumor microenvironment (TME), which refers to a part of the microbial community that exists inside the tumor and constitutes the TME. Studies have shown that intratumoral microbes promote cancer progression by affecting epithelial cells, tumor cells, and immune cells, and by promoting DNA damage, metabolic reprogramming, and the production of oncogenic non-coding RNAs. The effects of different intratumoral microorganisms on CRC are two-fold. They may either promote tumor growth or inhibit its progression. Intratumoral bacteria are able to remodel the immune response in the TME, suppress antitumor immunity, and promote immune escape (Xie et al., 2022). Intratumoral and intestinal flora are closely related but significantly different in composition and function. In terms of spatial distribution, intestinal flora is widely colonized in the intestinal mucosa and feces. In contrast, intratumoral bacteria are mainly enriched in hypovascularized areas of the tumor tissues or immunosuppressive microenvironments, which are in direct contact with cancer cells. In terms of function, intestinal flora remotely modulate systemic immune and inflammatory responses through metabolites, whereas intratumoral bacteria are directly involved in the tumor through local effects (e.g., activation of TLR4 signaling and the regulation of non-coding RNAs), cell proliferation, apoptosis, and metabolic reprogramming (Yang, 2024). There are complex interactions between the intestinal flora and intratumoral microorganisms. Gut flora can influence the composition and function of intratumoral microorganisms through metabolites (e.g., short-chain fatty acids) and immunomodulatory effects, which in turn affect CRC progression. For example, dysbiosis of intestinal flora may indirectly promote the proliferation and carcinogenesis of intratumoral microorganisms by altering the intestinal barrier function and immune microenvironment (Li et al., 2022). Although intratumoral flora has gradually gained attention in recent years, the study of intratumoral flora is still in its infancy compared with that of intestinal flora. Intratumoral flora and intestinal flora may have different interaction modes, but there is a lack of systematic comparative studies to reveal the differences and connections between them. Studies on the association between intratumoral and intestinal flora still face challenges, such as complex mechanisms, insufficient research, unclear causality, and limited technical means.

This study used 16S ribosomal RNA sequencing to compare gut microbiota between healthy individuals and CRC patients. The primary objectives were: (1) to identify differences in microbial diversity and composition between CRC patients and healthy individuals; (2) to assess the spatial heterogeneity of microbiota among cancerous tissue, paracancerous tissue, and feces within CRC patients; and (3) to explore potential associations between specific microbial signatures and key clinical tumor characteristics. Our findings aim to provide deeper insights into the spatial dynamics of CRC-associated microbiota and to evaluate its potential utility for diagnosis and targeted therapeutic strategies.

## 2 Materials and methods

### 2.1 Study subjects

Thirty-five patients with CRC diagnosed by pathology and requiring surgical treatment at the General Hospital of Ningxia Medical University from September 2023 to September 2024 were collected. Among these, five patients were excluded due to the presence of other primary malignant tumors and patient/family refusal to participate. Thirty patients were screened according to the inclusion and exclusion criteria. Thirty fecal samples were collected, and 60 samples were collected from CRC tissues and paracancerous tissues (5 cm from the cancerous tissues). Thirty stool samples were collected from healthy volunteers.

### 2.2 Inclusion and exclusion criteria

The inclusion criteria for CRC patients were the following: (1) patients with a clear histopathological diagnosis; (2) patients who had not received surgery, radiotherapy, chemotherapy, or immunotherapy; and (3) patients who had signed the informed consent.

The exclusion criteria for CRC patients were the following: (1) having inflammatory bowel disease; (2) Received chemotherapy within 2 weeks before sampling; (3) Used antibiotics/probiotics within 2 weeks before sampling. The inclusion criteria for healthy volunteers were as follows: (1) no infectious diseases; and (2) no history of special diets. The exclusion criteria for the healthy volunteers were as follows: (1) with family history of colorectal adenoma or CRC. (3) Used antibiotics/probiotics within 2 weeks before sampling. The study was approved by the ethics review committee (Approval No.KYLL-2025-1827), and all study subjects signed an informed consent form.

### 2.3 Sample collection

Stool samples: The subject should first empty their bladder and flush the toilet to prevent urine contamination. Place a small basin lined with tissue paper into the toilet. Defecate directly into the center of the basin. Using the collection spoon, stir the stool thoroughly from the middle section and scoop out approximately 5 grams (filling the collection box to 2/3 capacity). Immediately tighten the box lid. Place the collection box into a resealable bag, transfer it promptly into a dry ice container, and store it in a -80°C freezer within 24 hours.

Tumor samples: Tumor tissue specimens must be collected immediately upon surgical resection; the interval from tissue devitalization to the initiation of processing should not exceed 30 min to minimize ischemia- and metabolism-induced alterations of the microbial community. To prevent RNA degradation and changes in the microbial community, the samples were immersed in an RNA stabilizer (RNAlater) and stored frozen at -80°C.

### 2.4 Measurement of clinical indicators

Initial blood test data were collected from the CRC patients after admission to the hospital. This included a white blood cell (WBC) count, hemoglobin (Hb), relative ratio of neutrophils to lymphocytes, blood urea nitrogen (BUN), serum creatinine (Scr), aspartate aminotransferase (AST), alanine aminotransferase (ALT), total bilirubin (TBIL), albumin (ALB), alkaline phosphatase (AKP), gamma-glutamyltransferase (GGT), carcinoembryonic antigen (CEA), and glycoantigen 199 (CA199), as well as the pathologic Tumor, Node, Metastasis(TNM) stage of the resected tumors, number of lymph node metastases, and size of the tumors.

### 2.5 Microbiome total DNA extraction and detection

Total DNA of the microbial community was extracted from the fecal samples of the CRC patients and healthy volunteers according to the instructions of the FastPure Stool DNA Isolation Kit (MJYH, Shanghai, China). The integrity of the DNA was detected using 1% agarose gel electrophoresis, and DNA concentration and purity were determined using the NanoDrop2000 (Thermo Fisher Scientific, USA).

### 2.6 PCR amplification

The above extracted DNA was used as a template, and the upstream primer 338F (5’-ACTCCTACGGGGAGGCAGCAG-3’) and downstream primer 806R (5’-GGACTACHVGGGTWTCTAAT-3’), which carried the barcode sequence, were used. PCR amplification of the V3–V4 variable region of the 16S rRNA gene was carried out as follows: pre-denaturation at 95°C for 3 min, 29 cycles of denaturation at 95°C for 30 s, annealing at 53°C for 30 s, and extension at 72°C for 45 s, followed by a stable extension at 72°C for 10 min with storage at 10°C. The PCR instrument was an ABI GeneAmp^®^ Model 9700. The PCR reaction consisted of 10 μL 2×Pro Taq, 0.8 μL (5 μM) forward primer, 0.8 μL (5 μM) reverse primer, and 10 ng/μL template DNA. The reaction mixture was supplemented with ddH2O to 20 μL. Three replicates were performed for each sample. PCR products from the same samples were mixed and analyzed by 2% agarose gel electrophoresis to detect fragment size. The target bands were excised, gel-purified on 2% agarose gels, and quantified using a Synergy HTX (Biotek, USA).

### 2.7 Sequencing library construction

The purified PCR products were used for library construction using the NEXTFLEX Rapid DNA-Seq Kit (Bioo Scientific, Austin, Texas, USA): (1) junction linkage; (2) removal of junction self-linkage by magnetic bead screening; (3) enrichment of the library template by PCR amplification; and (4) recovery of the PCR products by magnetic bead recycling to obtain the final library. The second-generation sequencing was performed using the NextSeq 2000 PE300 platform from Illumina (Shanghai Meiji Biomedical Technology Co., Ltd.), with an average Q30 value of 96.6% for the raw data. The third-generation sequencing uses Pacbio. Although Q30 is not used as a base quality assessment, the raw data has achieved high accuracy in obtaining Hifi reads after quality control using SMRT Link 11.0 software.

### 2.8 Sequencing on the illumina platform

The second-generation sequencing was performed using the Illumina NextSeq 2000 platform (PE300) at Shanghai Majorbio Bio-pharm Technology Co., Ltd. The detailed procedure is as follows:

1. One end of the DNA fragment was complementary to an immobilized adaptor on the flow cell and fixed in place.
2. The other end randomly bound to a nearby immobilized adaptor via complementary base pairing, forming a “bridge.”
3. Bridge PCR amplification was carried out to generate DNA clusters.
4. DNA amplicons were linearized into single strands.
5. Engineered DNA polymerase and four fluorescently labeled dNTPs were added, allowing only one base to be incorporated per synthesis cycle.
6. The flow cell surface was scanned with a laser to detect the fluorescence signal corresponding to the nucleotide incorporated in each template strand during the first cycle.
7. The fluorophore and the blocking terminator were chemically cleaved, restoring the 3′-end reactivity for incorporation of the next nucleotide.
8. Fluorescence signals collected in each cycle were analyzed to determine the sequence of the template DNA fragments.

### 2.9 Sequencing on the Pacbio

The third-generation sequencing uses Pacbio. Purified products of PCR were pooled in equimolar and DNA library was constructed using the SMRTbell prep kit 3.0 (Pacifc Biosciences, CA, USA) according to PacBio’s instructions. Purified SMRTbell libraries were sequenced on the Pacbio Sequel IIe System (Pacifc Biosciences, CA, USA) by Majorbio Bio-Pharm Technology Co. Ltd. (Shanghai, China). PacBio raw reads were processed using the SMRTLink analysis software (version 11.0) to obtain high-quality Hifi reads with a minimum of three full passes and 99% sequence accuracy. Hifi reads were barcode-identified and length-filtered. For bacterial 16S rRNA gene, sequences with a length < 1,000 or >1,800 bp were removed. The Hifi reads were de-noised using DADA2 (Callahan et al., 2016) plugin in the Qiime2 (Bolyen et al., 2019) (version 2020.2) pipeline with recommended parameters, which obtains single nucleotide resolution based on error profiles within samples. DADA2 denoised sequences are usually called amplicon sequence variants (ASVs). To minimize the effects of sequencing depth on alpha and beta diversity measure, the number of sequence from each sample was rarefied to 6004 which still yielded an average Good’s coverage of 99.90%. Taxonomic assignment of ASVs was performed using the classify-consensus-blast(Blast) consensus taxonomy classifier implemented in Qiime2 and the NT taxon -core -16s database (v2024).

### 2.10 ASV denoising

After demultiplexing, the resulting sequences were quality filtered with fastp (v0.19.6) (Chen et al., 2018) and merged with FLASH (v1.2.11) (Magoč and Salzberg, 2011). Then the high-quality sequences were denoised using DADA2 (Callahan et al., 2016) plugin in the Qiime2 (Bolyen et al., 2019) (version 2020.2) pipeline with recommended parameters, which obtains single nucleotide resolution based on error profiles within samples. DADA2denoised sequences are usually called amplicon sequence variants (ASVs). To minimize the effects of sequencing depth on alpha and beta diversity measure, the number of sequence from each sample was rarefied to 20937, which still yielded an average Good’s coverage of 99.90%. Taxonomic assignment of ASVs was performed using the Naive bayes consensus taxonomy classifier implemented in Qiime2 and the SILVA 16S rRNA database (v138) using confidence threshold of 0.7.

### 2.11 Statistical analysis

All data analyses were performed on the Meggie BioCloud platform (https://cloud.majorbio.com). Alpha diversity indices, including species richness (Sobs), Shannon diversity, and Simpson indices, were calculated using mothur software (http://www.mothur.org/wiki/Calculators, version 1.30.2). Differences in alpha diversity between groups were assessed using the Wilcoxon rank-sum test. Principal coordinate analysis (PCoA) based on the Bray-Curtis distance algorithm was used to test the similarity of the microbial community structure among the samples. Common/endemic core species were obtained using Venn diagram analysis. A non-parametric Wilcoxon signed-rank test was used to assess differences in paired samples. Species were selected for correlation network diagram analysis based on Spearman correlation |*r*| > 0.6, *p* < 0.05. The Gut Microbiota Health Index (GMHI) is calculated using Python (version 2.7.10) software and the following formula.

Calculation formula:


$$h_{i,M_{H},M_{N}}=\log_{10}(\frac{\frac{R_{M_{H}}}{\left|M_{H}\right|}\sum_{j\in M_{H}}|n_{j}\log(n_{j})|}{\frac{R_{M_{N}}}{\left|M_{N}\right|}\sum_{j\in M_{N}}|n_{j}\log(n_{j})|})$$

(Gupta et al., 2020).

The Microbial Dysbiosis Index (MDI) was calculated using the R vegan package (version 2.4.3) in R (version 3.3.1) and Python (version 2.7.10), and is defined as: MDI=log10[(total abundance in genera increased in disease group)/(total abundance in genera decreased in disease group)] (Gunathilake et al., 2020).

## 3 Results

### 3.1 Alpha diversity analysis

**Figure 1:**
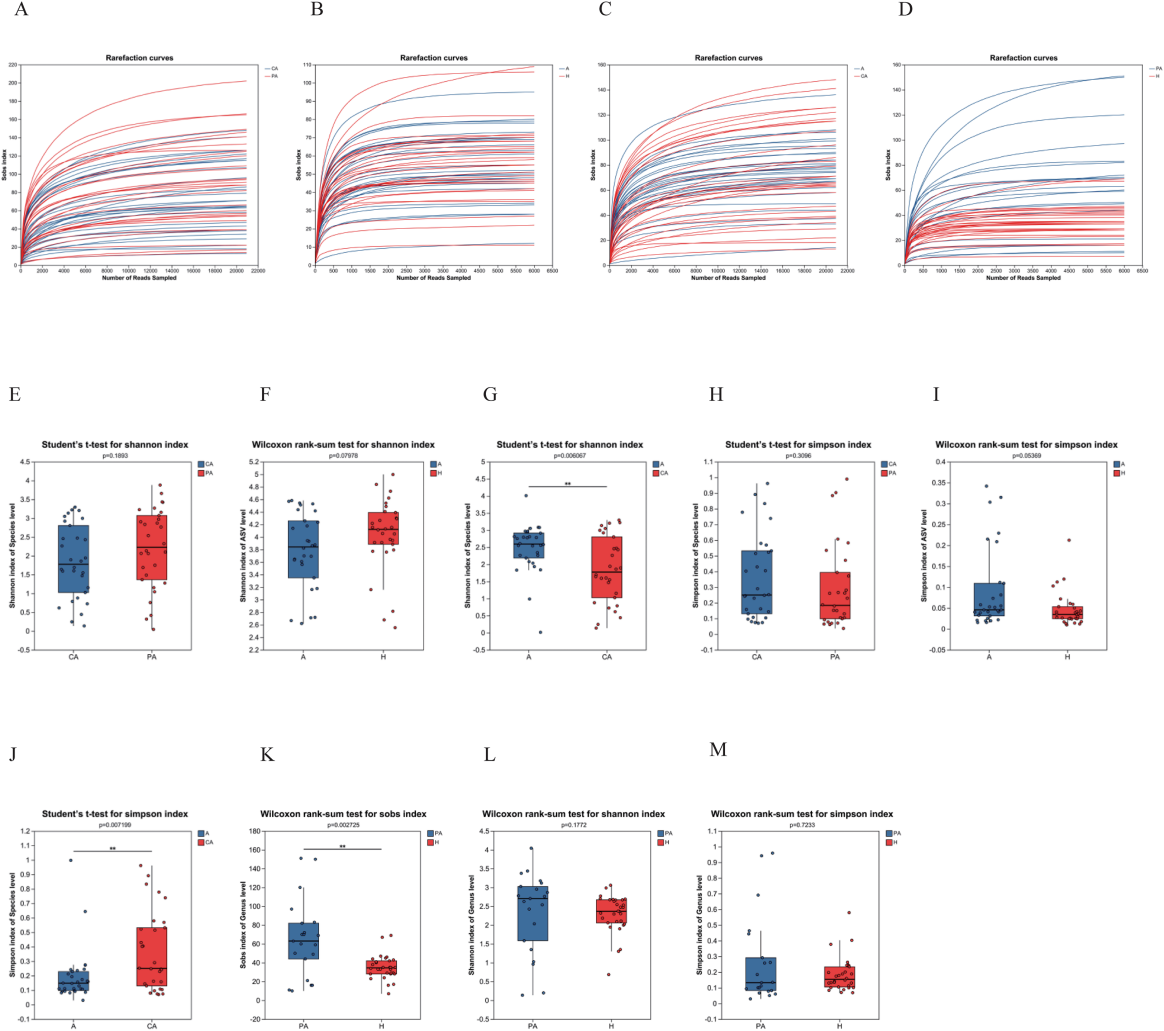
Alpha diversity analysis map. **(A–D)** The rarefaction curves of the Sobs index at the genus level. **(E–M)** Alpha diversity index plots. CA, cancerous tissue samples; PA, paracancerous tissue samples; A, fecal samples from CRC patients; H, fecal samples of healthy individuals), and the vertical axis represents the corresponding indices. * represents *p* < 0.05, ** represents *p* < 0.01, and *** represents *p* < 0.001.

In this study, the dilution curves (**Figures 1A–D**) illustrate a gradual increase in species richness with an increase in the number of sequences, which eventually leveled off. This indicates a gradual attainment of stability in species diversity, suggesting that the amount of experimental sequencing data was reasonable. Additionally, it implies that a greater amount of data yields diminishing returns in the discovery of new species. Alpha diversity serves as a measure of the abundance and diversity of microbiota within samples. In this study, the observed richness (Sobs),the Shannon diversity index and the Simpson diversity index(For the Simpson diversity index (D), smaller values indicate higher diversity.) were utilized for evaluation. For the tissue samples, the Shannon index was higher in the paracancerous tissue compared to the cancerous tissue (**Figure 1E**). Conversely, the Simpson index showed the opposite trend (**Figure 1H**). Although the differences were not statistically significant, there was a tendency for the paracancerous tissue to exhibit higher alpha diversity than the cancerous tissue.

The Shannon index was lower in the fecal samples from patients with CRC compared to healthy individuals (**Figure 1F**). Conversely, the Simpson index showed the opposite trend (**Figure 1I**). While these differences were not statistically significant, the alpha diversity tended to be greater in healthy individuals than in individuals with CRC.

Comparing tissue and fecal samples from patients with CRC, the Shannon index was lower in the cancerous tissue samples than in the fecal samples (**Figure 1G**). This indicates that the diversity of the community was higher in the fecal samples than in the cancerous tissue samples from the same CRC patients. Similarly, the Simpson index was higher in the cancerous tissue samples than in the fecal samples, suggesting that the diversity of the fecal microbiota was greater than that of colorectal cancer (CRC) tissues (**Figure 1J**). This difference was statistically significant (*p* < 0.01), suggesting that the presence of dominant species in the feces from CRC patients reduced the Simpson index.

For paracancerous tissue samples from CRC patients and fecal samples from healthy individuals, the Sobs index was higher in the paracancerous tissue samples than in the fecal samples. This indicates that paracancerous tissues in CRC exhibited a higher richness compared to feces (**Figures 1K–M**). The difference was statistically significant in the Sobs index (*p* < 0.01).

### 3.2 Beta diversity analysis

#### 3.2.1 Sample-level cluster analysis

**Figure 2:**
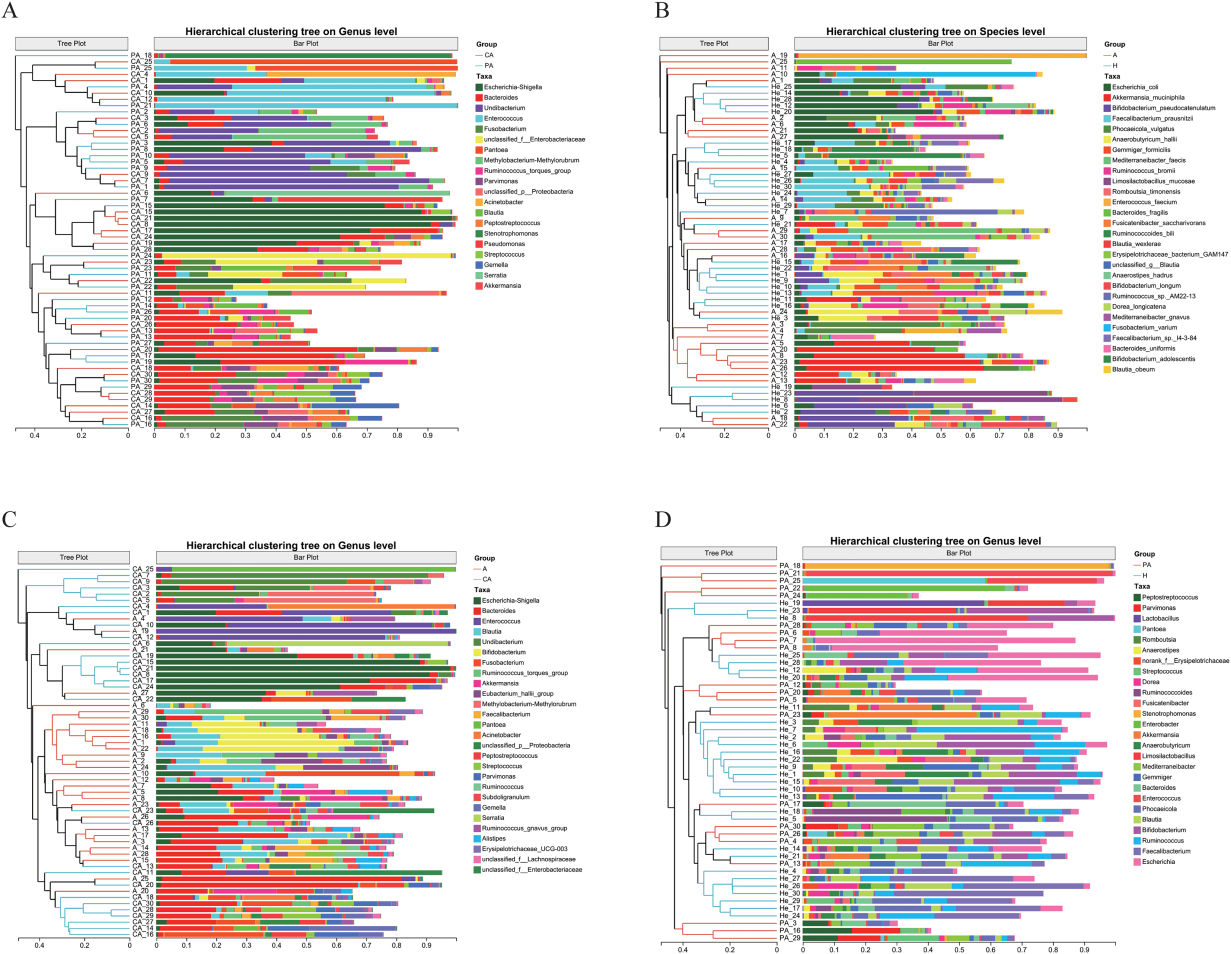
Hierarchical cluster analysis diagram. **(A)** CA vs. PA at genus level, **(B)** A vs. H at species level, **(C)** A vs. CA at genus level, **(D)** PA vs. H at genus level. The horizontal axis represents relative abundance, with values ranging from 0 to 1. Larger values indicate a higher abundance of the genus or species in the sample. The vertical axis is a hierarchical clustering tree, with closer branching indicating a more similar distribution pattern of the genera or species in the sample. Bars of different colors represent the abundance of different microbial genera, and the genus or species name corresponding to each color is listed in the legend. CA: cancerous tissue samples; PA: paracancerous tissue samples; A: fecal samples from CRC patients; H: fecal samples of healthy individuals.

Sample-level cluster analysis revealed similarities and differences in the microbial community structure across the samples. **Figure 2A** shows that certain CRC tissue samples clustered together while paracancerous tissue samples clustered in another section. This indicates that the cancerous and paracancerous tissues differed in microbial community structure. In **Figure 2A**, cancerous tissue samples and paracancerous tissue samples formed relatively independent branches in the clustering tree, further confirming differences in microbial community structure between the two sample types. In the CRC samples, some samples were distantly clustered from other cancerous tissue samples, indicating some heterogeneity in the microbial community even in the same sample type. This heterogeneity was also observed in the paracancerous tissue samples, suggesting that the composition of the microbial communities in paracancerous tissues is not homogeneous. For the fecal samples, there were differences in the microbial community structure between CRC patients and healthy individuals (**Figure 2B**). Similarly, microbial community differences existed in the fecal samples and tissue samples from CRC patients, and in paracancerous tissues from CRC patients and fecal samples from healthy individuals (**Figures 2C, D**).

**Figure 2** also shows that certain flora were more abundant in specific sample groups. For example, *Escherichia-Shigella* showed higher abundance in cancerous tissues than in paracancerous tissues. *Escherichia coli* and *Akkermansia muciniphila* showed higher abundance in CRC patients’ feces than in feces from healthy individuals. *Escherichia-Shigella*, *Bacteroides*, *Enterococcus*, and other genera showed higher abundance in CRC tissues than in feces.

#### 3.2.2 Principal coordinate analysis

**Figure 3:**
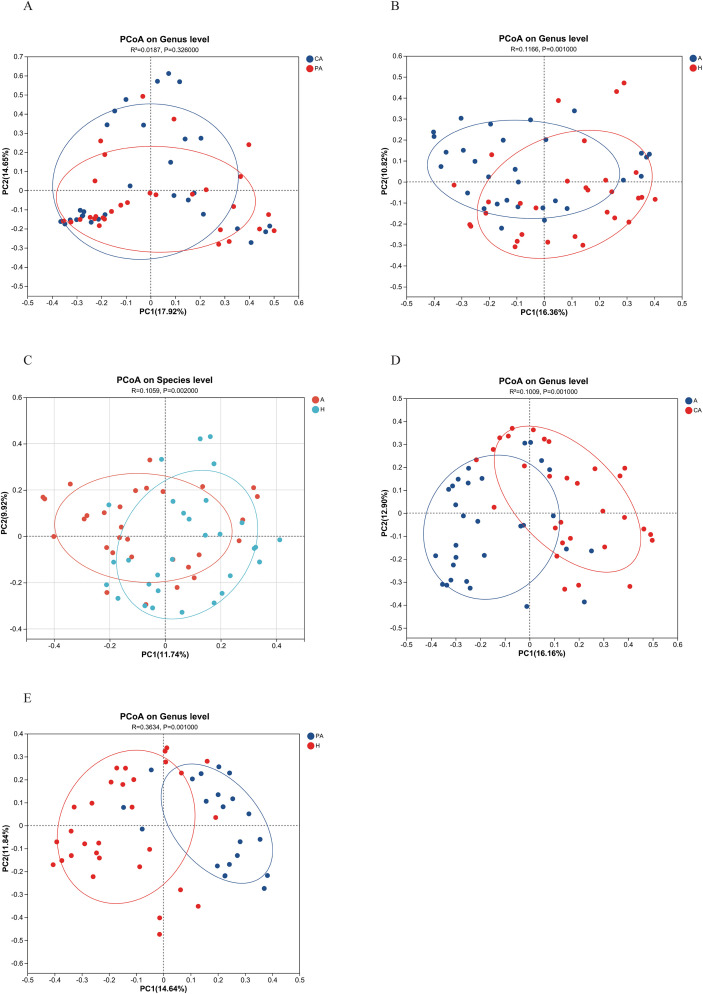
PCoA analysis diagram. **(A)** CA vs. PA at genus level, **(B)** A vs. H at genus level, **(C)** A vs. H at species level, **(D)** A vs. CA at genus level, (E) PA vs. H at genus level. The horizontal axis represents PC1 (principal coordinate 1), the vertical axis represents PC2 (principal coordinate 2), and the ellipses represent the 95% confidence intervals for each group to show the distribution range of samples within the group. CA: cancerous tissue samples; PA: paracancerous tissue samples; A: fecal samples from CRC patients; H: fecal samples of healthy individuals.

In this study, colony beta diversity was assessed by PCoA combined with Bray-Curtis differences. Statistical significance was analyzed and plotted by PCoA statistics in R language (version 3.3.1). For the tissue samples (**Figure 3A**), there was some separation between CRC tissues and paracancerous tissues on PC1 and PC2. This indicated a difference in microbial community structure between the cancerous tissues and paracancerous tissues (*R^2^* = 0.0187, *p* = 0.326000). However, although there was some difference between the two groups, this difference was not statistically significant (*p*>0.05). For the fecal samples (**Figures 3B, C**), the gut microbiota of the CRC patients was clearly separated from that of the healthy individuals at both genus and species levels, with statistically significant differences in bacterial flora beta diversity (*p* < 0.01). In addition, significant structural differences in beta diversity were observed between the fecal samples of CRC patients and the patient’s corresponding cancerous tissue samples (**Figure 3D**). Paracancerous tissues from CRC patients also showed significant beta diversity differences compared to fecal samples from healthy individuals (*p* < 0.01) (**Figure 3E**).

### 3.3 Colony characterization index

#### 3.3.1 Gut microbiome health index and gut microbiome dysbiosis index

**Figure 4:**
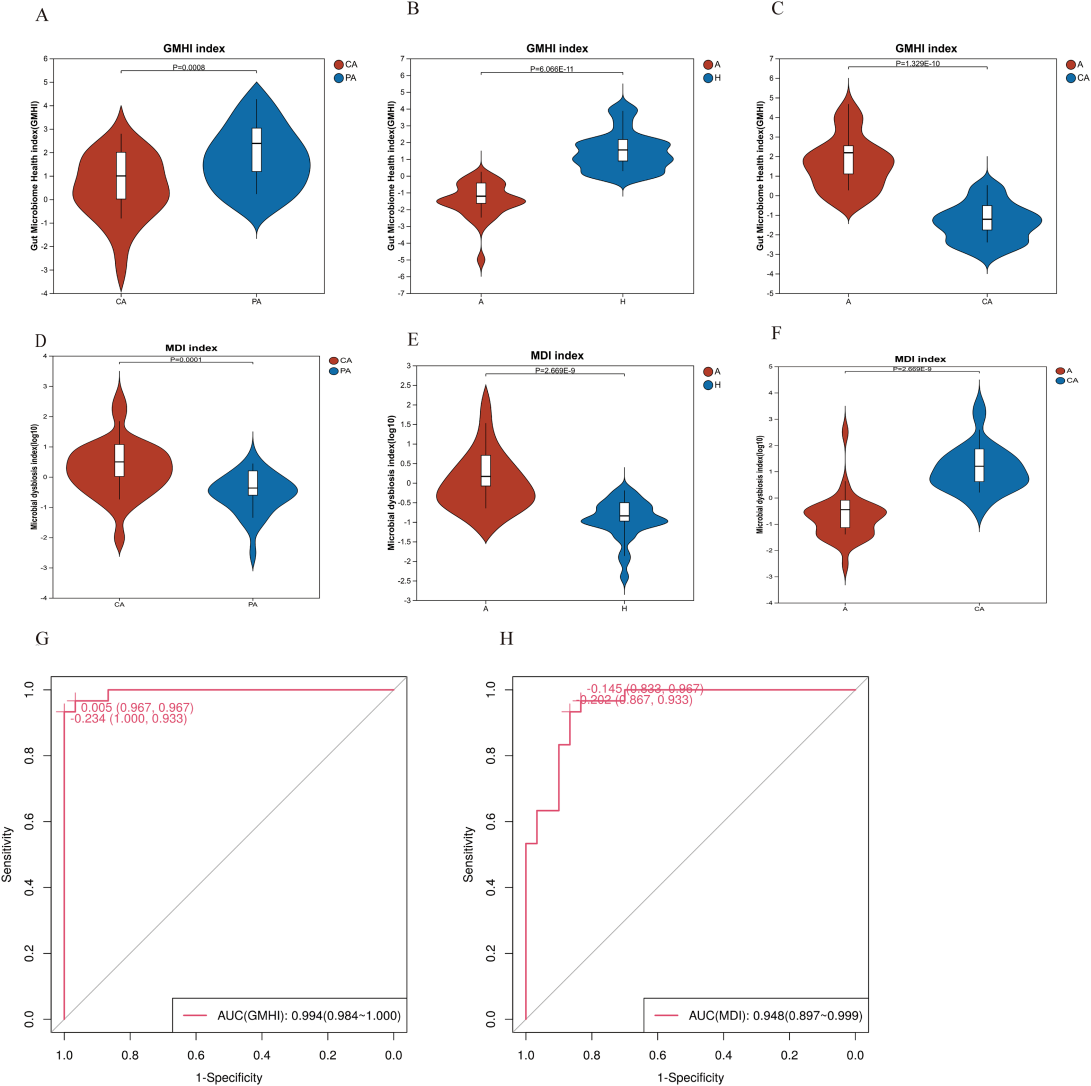
GMHI and MDI. Panels **(A–C)** are GMHI plots; **(D–F)** are MDI plots. The horizontal axis represents different sample groups, and the vertical axis represents the GMHI index and MDI index. CA: cancerous tissue samples; PA: paracancerous tissue samples; A: fecal samples from CRC patients; H: fecal samples of healthy individuals. **(G, H)** ROC curves of GMHI versus MDI.Horizontal axis: 1-specificity (false-positive rate); vertical axis: sensitivity (true-positive rate).

By analyzing differences in the Gut Microbiome Health Index (GMHI) of different sample groups, it was found that the microbiota of paracancerous tissues had significantly higher GMHIs and were in better health than that of cancerous tissues, with a statistically significant difference (*p* < 0.001) (**Figure 4A**). In addition, healthy individuals had significantly better intestinal health compared to patients with CRC (*p* < 0.001)(**Figure 4B**). Among the CRC patients, the fecal samples had significantly higher GMHI values than the cancerous tissue samples (*p* < 0.001) (**Figure 4C**).

Differential analysis of the Microbiome Dysbiosis Index (MDI) in different sample groups revealed that the microbiota of cancerous tissues had significantly (*p* < 0.001) higher dysbiosis indexes than the microbiota of paracancerous tissues (**Figure 4D**), indicating a greater degree of dysbiosis. The degree of dysbiosis of the intestinal flora of patients with CRC was greater than that of healthy individuals. The difference was statistically significant (*p* < 0.001) (**Figure 4E**). Cancerous tissue samples from CRC patients had a significantly higher MDI than fecal samples (*p* < 0.001) (**Figure 4F**). The GMIH index achieved an AUC of 99.4% (95% CI: 98.4%-100%) in distinguishing CRC patients from healthy controls, while the MDI index yielded an AUC of 94.8% (95% CI: 89.7%-99.9%)(**Figures 4G, H**).

### 3.4 Community composition analysis

#### 3.4.1 Species Venn diagram analysis

**Figure 5:**
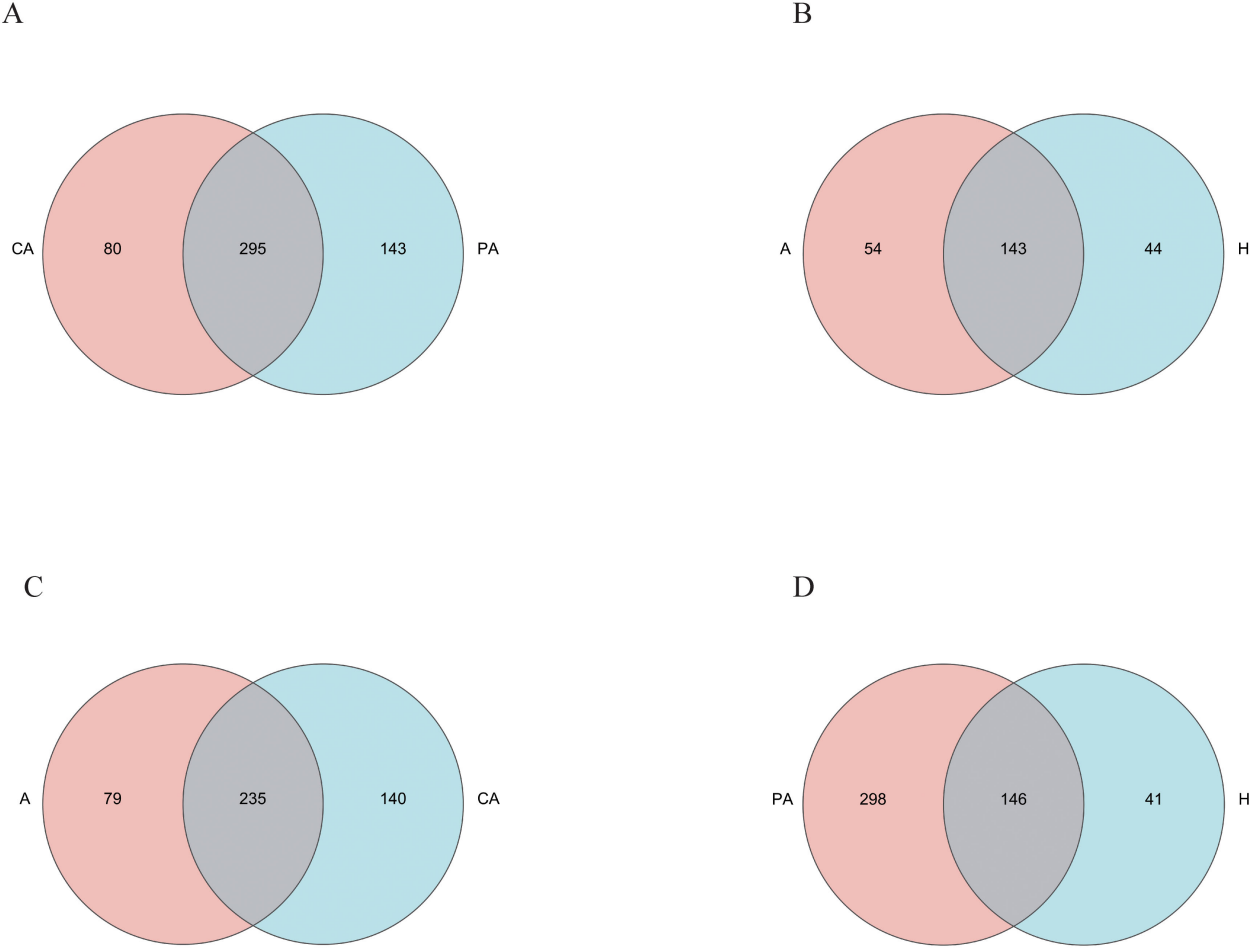
Venn diagram analysis of species. **(A)** CA vs. PA; **(B)** A vs. healthy controls (H); **(C)** A vs. CA; **(D)** PA vs. H. CA represents the cancerous tissues of CRC patients, PA represents the paracancerous tissues of the CRC patients, A represents the fecal samples of the CRC patients, and H represents the fecal samples of healthy individuals.

Based on the genus level, a total of 80 species were found only in the cancerous tissues of the CRC patients, a total of 143 species were found only in paracancerous tissues, and a total of 295 species were found in both cancerous and paracancerous tissues (**Figure 5A**). The total number of species in the cancerous tissues (CA) was 375 (80 endemic species + 295 species in common). The total number of species in the paracancerous tissues (PA) was 438 (143 endemic species + 295 species in common). Based on the genus level, a total of 54 species was found only in the feces of the CRC patients and a total of 44 species was found only in the feces of the healthy individuals, for a total of 197 species in the feces of the CRC patients and 187 in the feces of the healthy individuals (**Figure 5B**). Based on the genus level, a total of 79 species was found only in the fecal samples of the CRC patients and a total of 140 species was found only in the cancerous tissues of CRC patients, and a total of 235 species were found in both feces and cancerous tissues of CRC patients (**Figure 5C**). Based on the genus level, a total of 298 species was found only in the paracancerous tissues of CRC patients, a total of 41 species was found only in the feces of healthy individuals, and a total of 146 species was found in both fecal and cancerous tissue samples of CRC patients (**Figure 5D**). This difference in species distribution may reflect significant differences in microbial community structure between cancerous and paracancerous tissues, CRC patients and healthy individuals, and CRC patients’ feces and cancerous tissues.

#### 3.4.2 Community bar graph, community heatmap, and community circos

**Figure 6:**
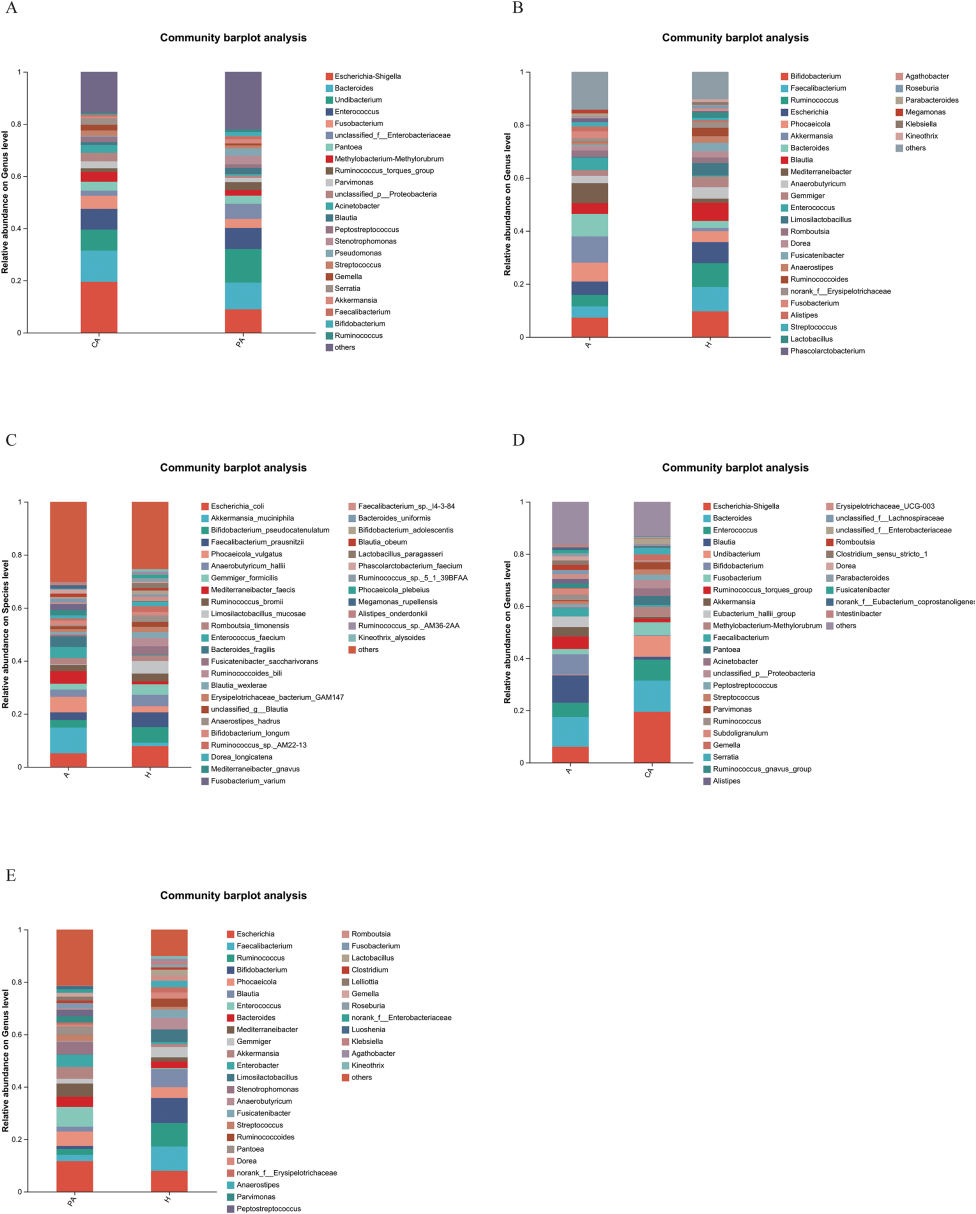
Bar graph of the community. **(A)** CA vs. PA at genus level, **(B)** A vs. H at genus level, **(C)** A vs. H at species level, **(D)** A vs. CA at genus level, **(E)** PA vs. H at genus level. The horizontal axis represents the different sample groups, and the vertical axis represents the relative abundance at a taxonomic level ranging from 0 to 1, which indicates the relative proportion of the samples at that taxonomic level. Each bar represents a genus or species, and the height of the bar indicates the sum of the relative abundance of all microbial genera or species in that sample. CA: cancerous tissue samples; PA: paracancerous tissue samples; A: fecal samples from CRC patients; H: fecal samples of healthy individuals.

**Figure 7:**
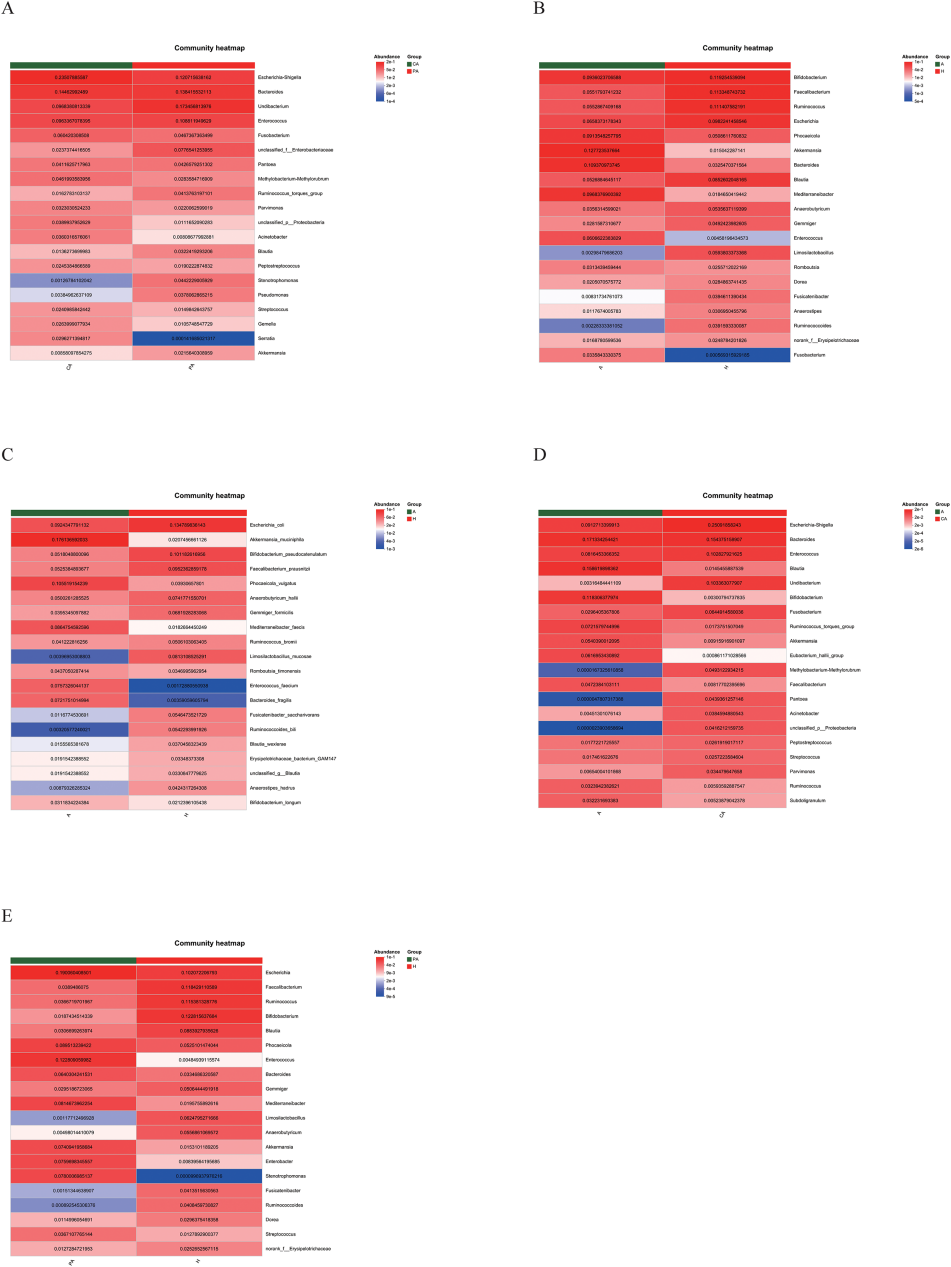
Heatmap diagram of the community. **(A)** CA vs. PA at genus level, **(B)** A vs. H at genus level, **(C)** A vs. H at species level, **(D)** A vs. CA at genus level, **(E)** PA vs. H at genus level. The horizontal axis represents different sample groups, and the vertical axis represents the different bacterial genus names or species names. The value next to the colored bar in the upper right corner indicates the logarithmic value of abundance. Red indicates high abundance, blue indicates low abundance, and white indicates medium abundance. CA: cancerous tissue samples; PA: paracancerous tissue samples; A: fecal samples from CRC patients; H: fecal samples of healthy individuals.

**Figure 8:**
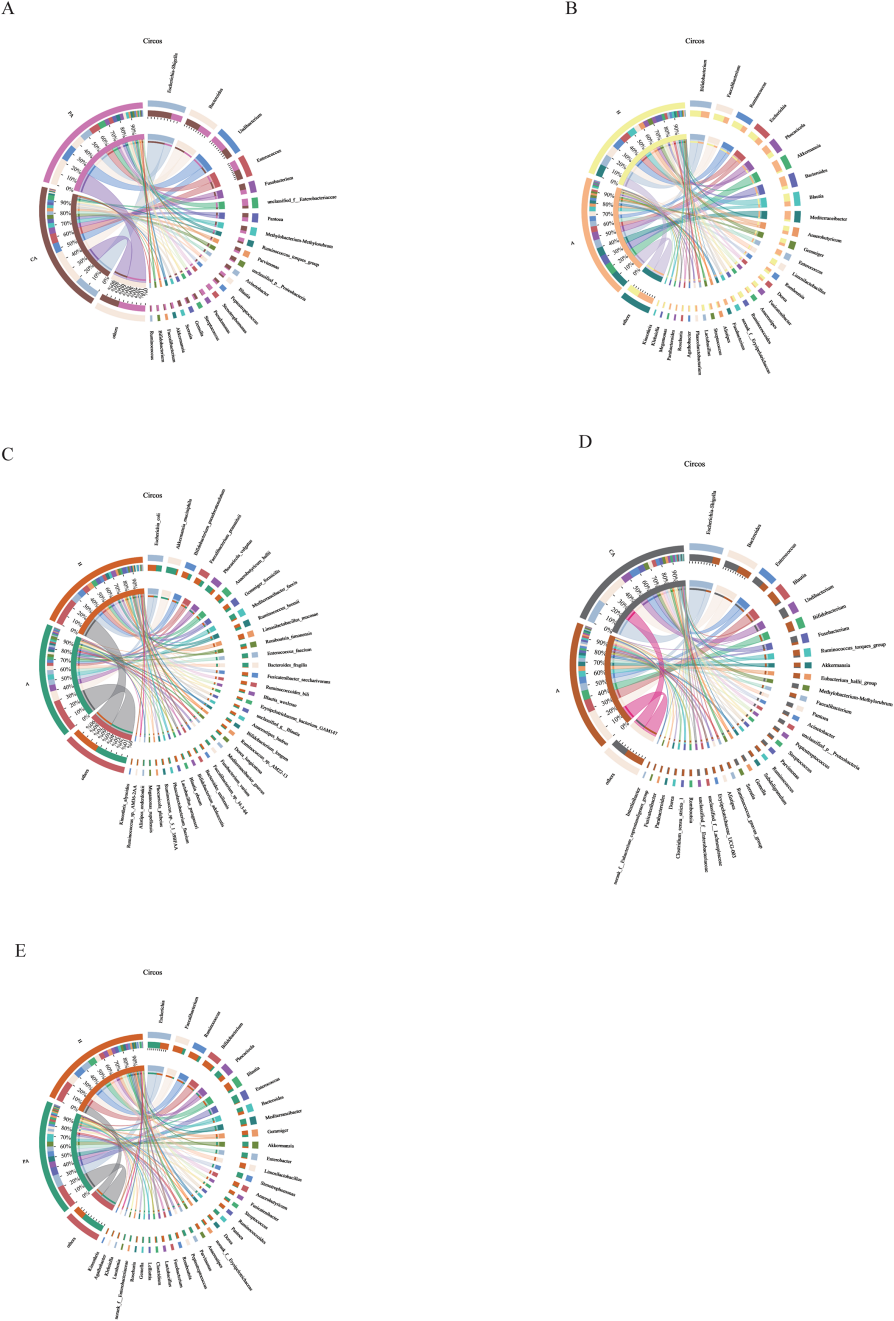
Circos diagram of the community. **(A)** CA vs. PA at genus level, **(B)** A vs. H at genus level, **(C)** A vs. H at species level, **(D)** A vs. CA at genus level, **(E)** PA vs. H at genus level. The outer circle represents the different sample groups, and the inner circle is a circular bar graph showing the abundance of different microbial taxa in the sample. Each bar represents a microbial taxon, and the length of the bar indicates the relative abundance of that taxon in the sample. Connecting lines: Lines extending from the bars in the inner circle connect to the sample groups in the outer circle, indicating the distribution of these microbial taxa in the corresponding samples, and the thickness of the lines indicates the level of abundance. CA: cancerous tissue samples; PA: paracancerous tissue samples; A: fecal samples from CRC patients; H: fecal samples of healthy individuals.

In this study, the community composition of all taxa was analyzed at the genus and species levels. At the genus level, *Escherichia-Shigella*, *Bacteroides*, and *Enterococcus* showed high abundance in both cancerous and paracancerous tissues from CRC patients. However, *Escherichia-Shigella* and *Bacteroides* had higher relative fractions in cancerous tissues, while *Methylobacterium/Methylorubrum*, *Ruminococcus torques*, and *Blautia* had higher relative abundance in paracancerous tissues (**Figures 6A**, **7A**, **8A**).

At the genus level, a comparison of the bar graphs showing fecal sample composition revealed differences in the distribution of bacterial genera between CRC patients and healthy individuals (**Figures 6B**, **7B**, **8B**). The fecal samples from healthy individuals had a higher relative abundance of *Bifidobacterium*, *Faecalibacterium*, *Ruminococcus*, *Escherichia*, *Blautia*, and *Limosilactobacillus* than the fecal samples from CRC patients. In contrast, the fecal samples from CRC patients had a higher relative abundance of *Phocaeicola*, *Akkermansia*, *Bacteroides*, *Mediterraneibacter*, and *Enterococcus*.

At the species level, *Escherichia coli*, *Bifidobacterium pseudocatenulatum*, and *Limosilactobacillus mucosae* were dominant in healthy individuals. Meanwhile, *Akkermansia muciniphila*, *Phocaeicola vulgatus*, *Mediterraneibacter faecis*, *Enterococcus faecium*, and *Bacteroides fragilis* had a higher relative abundance in the fecal samples from CRC patients (**Figures 6C**, **7C**, **8C**). At the genus level, *Escherichia-Shigella*, *Bacteroides*, *Enterococcus*, and *Fusobacterium* had a higher relative abundance in the CRC tissue samples, and *Blautia*, *Bifidobacterium*, *Ruminococcus torques*, *Akkermansia*, and *Faecalibacterium* had a higher relative abundance in the fecal samples than the cancerous tissue samples from CRC patients (**Figures 6D**, **7D**, **8D**). At the genus level, *Escherichia*, *Enterococcus*, *Bacteroides*, *Mediterraneibacter*, and *Akkermansia* had a higher abundance in paracancerous tissues from the CRC patients than in fecal samples from healthy individuals. *Faecalibacterium*, *Ruminococcus*, *Bifidobacterium*, and *Limosilactobacillus* were in high abundance in fecal samples from healthy individuals (**Figures 6E**, **7E**, **8E**).

### 3.5 Analysis of species differences

**Figure 9:**
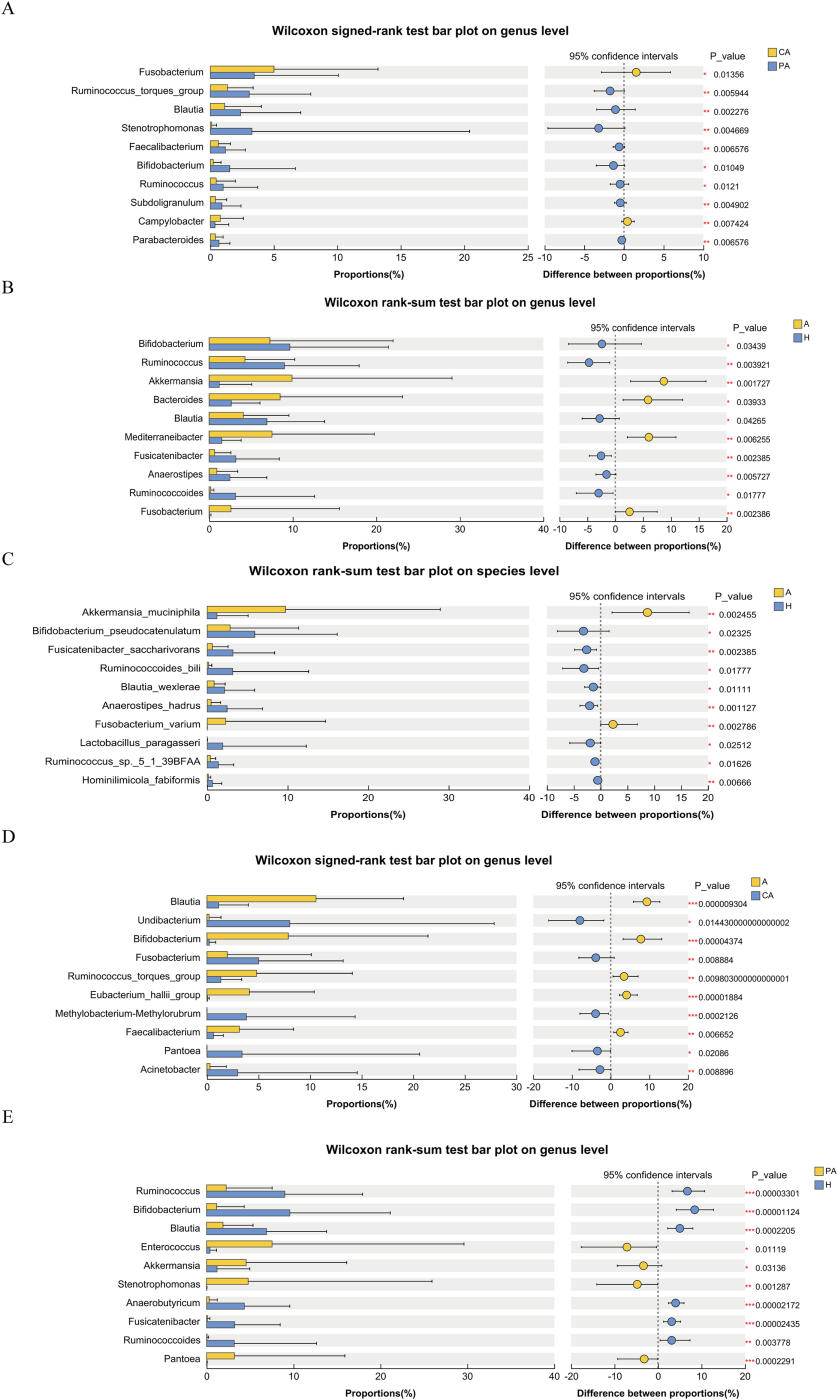
Analysis of species differences. **(A)** CA vs. PA at genus level, **(B)** A vs. H at genus level, **(C)** A vs. H at species level, **(D)** A vs. CA at genus level, **(E)** PA vs. H at genus level. Horizontal axis: the left side indicates the percentage of each microbial genus or species in the sample, the right side indicates the percentage difference in relative abundance of each microbial genus or species from -25% to 25%, indicating the direction and magnitude of the difference. The vertical axis indicates the name of each microbial genus or species. CA: cancerous tissue samples; PA: paracancerous tissue samples; A: fecal samples from CRC patients; H: fecal samples of healthy individuals. On the far right are the *p*-values. * represents *p* < 0.05, ** represents *p* < 0.01, and *** represents *p* < 0.001.

Comparing differences in bacterial genera between cancerous and paracancerous tissues from CRC patients (**Table 1**), it was found that *Fusobacterium* had a significantly higher proportion in the cancerous tissue samples (*p* < 0.05). *Ruminococcus*, *Faecalibacterium*, *Bifidobacterium*, and *Blautia* had a significantly higher proportion in the paracancerous tissue samples than cancerous tissue samples (*p* < 0.05) (**Figure 9A**). Comparing feces from CRC patients and healthy individuals at the genus level (**Table 2**) revealed that *Bifidobacterium*, *Blautia*, and *Ruminococcus* accounted for a higher percentage in feces from healthy individuals, with a statistically significant difference between the cancerous and paracancerous tissue samples (*p* < 0.05). *Akkermansia*, *Bacteroides*, *Mediterraneibacter*, and *Enterococcus* accounted for a statistically higher proportion in the fecal samples from CRC patients than healthy individuals (*p* < 0.05) (**Figure 9B**). At the species level, fecal samples from CRC patients had a statistically higher percentage of *Akkermansia muciniphila* and *Fusobacterium varium* than the fecal samples of healthy individuals (*p* < 0.01) (**Figure 9C**). Comparing fecal and cancerous tissue samples from CRC patients (**Table 3**) at the level of analyzed genera revealed a significantly higher percentage of *Faecalibacterium*, *Bifidobacterium*, and *Blautia* in the fecal samples than cancerous tissue samples (*p* < 0.05). *Fusobacterium*, *Methylobacterium/Methylorubrum*, and *Acinetobacter* accounted for a higher percentage in the cancerous tissue samples in CRC patients, and the difference was statistically significant (*p* < 0.01) (**Figure 9D**). Comparing paracancerous tissues from CRC patients with feces from healthy patients (**Table 4**) at a genus level revealed that *Ruminococcus*, *Bifidobacterium*, *Blautia*, *Anaerobutyricum*, and *Fusicatenibacter* were in high abundance in feces from healthy individuals (**Figure 9E**). Paracancerous tissue samples from CRC patients had a high percentage of *Enterococcus*, *Akkermansia*, *Stenotrophomonas*, and *Pantoea* (*p* < 0.05).

**Table 1:** Differential Analysis Summary (CA and PA).

Species name	CA-mean(%)	CA-sd(%)	PA-mean(%)	PA-sd(%)	statistic(%)	P_value	Fold Change	P_adjust	Effectsize
g:Fusobacterium	5.008	8.193	3.466	6.614	353	0.01356	0.69209	0.3145	1.542
g:Ruminococcus_torques_group	1.349	2.021	3.069	4.84	74	0.00594	2.27502	0.2311	-1.753
g:Blautia	1.129	2.885	2.391	4.726	68.5	0.00228	2.1178	0.2251	-1.076
g:Stenotrophomonas	0.1051	0.3874	3.28	17.14	50.5	0.00467	31.20837	0.2311	-3.192
g:Faecalibacterium	0.6349	0.955	1.193	1.568	68	0.00658	1.87904	0.2311	-0.6083
g:Bifidobacterium	0.2336	0.6149	1.53	5.164	82	0.01049	6.54966	0.2991	-1.327
g:Ruminococcus	0.4609	1.512	1.016	2.721	23	0.0121	2.20438	0.3145	-0.491
g:Subdoligranulum	0.4068	0.8846	0.9049	1.51	45	0.0049	2.22443	0.2311	-0.466
g:Campylobacter	0.7886	1.817	0.3678	1.085	162	0.00742	0.4664	0.2395	0.4404
g:Parabacteroides	0.3975	0.6083	0.6771	0.8638	68	0.00658	1.7034	0.2311	-0.2778

**Table 2:** Differential analysis summary (A and H).

Species name	CA-mean(%)	CA-sd(%)	PA-mean(%)	PA-sd(%)	statistic(%)	P_value	Fold Change	P_adjust	Effectsize
g:Bifidobacterium	7.26	14.75	9.652	11.79	306.5	0.03439	0.75218	0.3658	-2.394
g:Ruminococcus	4.288	5.965	9.017	8.934	254.5	0.00392	0.47555	0.09174	-4.727
g:Akkermansia	9.907	19.15	1.218	3.879	660	0.00173	8.13383	0.08493	8.708
g:Bacteroides	8.483	14.66	2.634	3.453	589	0.03933	3.22058	0.3697	5.89
g:Blautia	4.087	5.458	6.901	6.916	312.5	0.04265	0.59223	0.3697	-2.813
g:Mediterraneibacter	7.511	12.25	1.495	2.323	635	0.00626	5.02408	0.1113	6.022
g:Fusicatenibacter	0.6451	1.956	3.194	5.19	248.5	0.00239	0.20197	0.08493	-2.55
g:Anaerostipes	0.9127	2.503	2.484	4.406	269.5	0.00573	0.36743	0.1113	-1.572
g:Ruminococcoides	0.1771	0.3973	3.17	9.441	315	0.01777	0.05587	0.2079	-2.994
g:Fusobacterium	2.605	12.98	0.04608	0.178	620	0.00239	56.53212	0.08493	2.559

**Table 3:** Differential analysis summary (A and CA).

Species name	CA-mean(%)	CA-sd(%)	PA-mean(%)	PA-sd(%)	statistic(%)	P_value	Fold Change	P_adjust	Effectsize
g:Blautia	10.56	8.497	1.129	2.885	423	9.304e-6	0.10691	0.00251	9.364
g:Undibacterium	0.2108	1.155	8.026	19.82	3	0.01443	38.074	0.1003	-7.941
g:Bifidobacterium	7.88	13.55	0.2336	0.6149	407	4.0E-5	0.02964	0.00377	7.802
g:Fusobacterium	1.974	8.139	5.008	8.193	96	0.00888	2.53698	0.07363	-3.841
g:Ruminococcus_torques_group	4.806	9.284	1.349	2.021	297	0.0098	0.28069	0.07945	3.467
g:Eubacterium_hallii_group	4.109	6.29	0.06687	0.1578	322	2.0E-5	0.01627	0.00251	4.121
g:Methylobacterium-Methylorubrum	0.00111	0.0061	3.829	10.53	0	0.00021	3437.16338	0.00689	-3.891
g:Faecalibacterium	3.146	5.245	0.6349	0.955	343.5	0.00665	0.20181	0.06134	2.544
g:Pantoea	0.00032	0.00174	3.412	17.21	1	0.02086	10716.0804	0.133	-3.425
g:Acinetobacter	0.3006	1.568	2.986	11.58	29.5	0.0089	9.93347	0.07363	-2.753

**Table 4:** Differential analysis summary (PA and H).

Species name	CA-mean(%)	CA-sd(%)	PA-mean(%)	PA-sd(%)	statistic(%)	P_value	Fold Change	P_adjust	Effectsize
g:Ruminococcus	8.996	8.943	2.249	5.289	532	3.0E-5	4	0.00222	6.747
g:Bifidobacterium	9.575	11.55	1.149	3.168	544.5	1.0E-5	8.33333	0.00139	8.426
g:Blautia	6.892	6.902	1.88	3.457	508	0.00022	3.66596	0.00794	5.012
g:Enterococcus	0.3781	0.7685	7.53	22.08	190	0.01119	0.05021	0.1263	-7.152
g:Akkermansia	1.194	3.774	4.543	11.57	203.5	0.03136	0.26282	0.1858	-3.349
g:Stenotrophomonas	0.00777	0.03236	4.783	21.14	193.5	0.00129	0.00163	0.03285	-4.775
g:Anaerobutyricum	4.342	5.229	0.3054	0.9237	529.5	2.0E-5	14.21742	0.00197	4.036
g:Fusicatenibacter	3.224	5.234	0.0928	0.2823	527	2.0E-5	34.74138	0.00197	3.131
g:Ruminococcoides	3.185	9.456	0.05473	0.1762	440	0.00378	58.19477	0.06979	3.13
g:Pantoea	0.0111	0.06082	3.195	12.71	176	0.00023	0.00347	0.00794	-3.183

Mean (%), Mean percentage of the species count; Sd (%), Standard deviation percentage of the species count; Statistic (%), Statistical metric, summarizing sample characteristics; p-value, Probability of observing a false positive result; a common threshold in statistical hypothesis testing where p < 0.05 indicates significant difference; Corrected p-value, Adjusted p-value; Effect size, quantifying the magnitude of the observed difference or association.

### 3.6 Clinical factor correlation analysis

**Figure 10:**
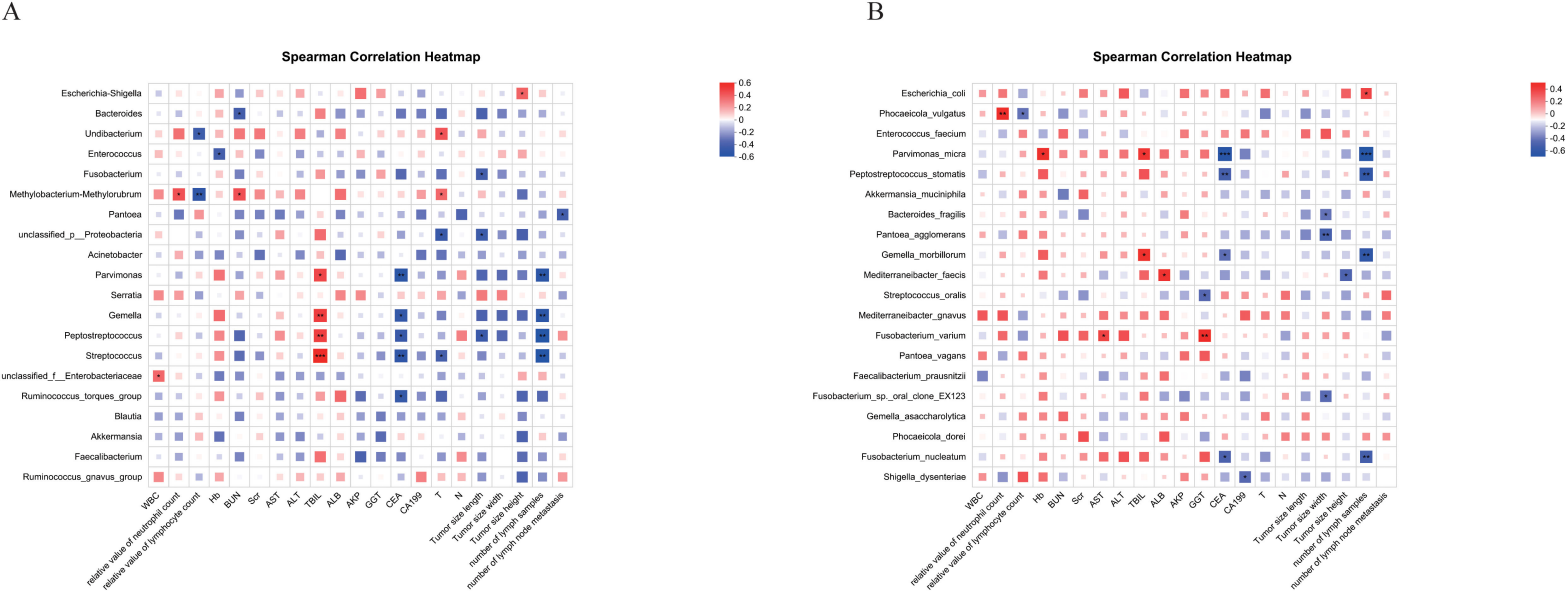
Clinical factor correlation analysis graph. **(A)** Analysis at the genus level; **(B)** Analysis at the species level: The correlation analysis graph between microbial genera and clinical parameters in CRC tissues. **(A)** genus level, and **(B)** species level. The horizontal axis represents clinical factors, and the vertical axis represents different microbial genera. Red indicates a positive correlation (value close to 1), blue indicates a negative correlation (value close to -1), and white indicates no significant correlation (value close to 0). * represents *p* < 0.05, ** represents *p* < 0.01, and *** represents *p* < 0.001.

By analyzing the correlation of different bacterial genera in cancerous tissues and clinical factors in CRC patients (**Table 5**), *Escherichia-Shigella*, for example, showed a statistically significant positive correlation with tumor size (*p* < 0.05). *Methylobacterium/Methylorubrum* showed a statistically significant positive correlation with tumor stage (*p* < 0.05). *Parvimonas*, *Gemella*, *Peptostreptococcus*, and *Streptococcus* were negatively correlated with the number of tumor lymph node metastases, and the difference was statistically significant (*p* < 0.01). *Parvimonas*, *Gemella*, *Peptostreptococcus*, and *Streptococcus* were negatively correlated with CEA values. The difference was statistically significant (*p* < 0.05). *Parvimonas*, *Gemella*, *Peptostreptococcus*, and *Streptococcus* were positively correlated with TBIL, and the difference was statistically significant (*p* < 0.05) (**Figure 10A**).

**Table 5:** Characteristics of study participants.

Characteristic	Total (N = 30)	colon (N = 10)	rectum (N = 20)	Statistic	p-value	p-adjust
**age (Mean ± SD)**	60.733 ± 10.619	61.600 ± 13.542	60.300 ± 9.200	t=0.274	0.78857	0.91999
**Gender**				χ2 = 0.820	0.36509	0.63891
female	14 (46.7%)	3 (30.0%)	11 (55.0%)			
male	16 (53.3%)	7 (70.0%)	9 (45.0%)			
**Nation**				χ2 = 2.145	0.34215	0.63891
the Han nationality	25 (83.3%)	8 (80.0%)	17 (85.0%)			
the Hui nationality	4 (13.3%)	1 (10.0%)	3 (15.0%)			
the Mongol nationality	1 (3.3%)	1 (10.0%)	0 (0%)			
**Family history**				χ2 = 3.333	0.06789	0.63891
no	30 (100.0%)	10 (100%)	20 (100%)			
**Smoking history**				χ2 = 0.021	0.88395	0.95194
no	22 (73.3%)	8 (80.0%)	14 (70.0%)			
yes	8 (26.7%)	2 (20.0%)	6 (30.0%)			
**Drinking history**				χ2 = 1.470	0.22535	0.63891
no	25 (83.3%)	10 (100%)	15 (75.0%)			
yes	5 (16.7%)	0 (0%)	5 (25.0%)			
**T**				χ2 = 2.531	0.28206	0.63891
2	2 (6.7%)	1 (10.0%)	1 (5.0%)			
3	12 (40.0%)	2 (20.0%)	10 (50.0%)			
4	16 (53.3%)	7 (70.0%)	9 (45.0%)			
**N**				χ2 = 3.750	0.15335	0.63891
0	15 (50.0%)	7 (70.0%)	8 (40.0%)			
1	10 (33.3%)	3 (30.0%)	7 (35.0%)			
2	5 (16.7%)	0 (0%)	5 (25.0%)			
**Number of lymph node metastasis (Median[Q1,Q3])**	1.000(0.000,2.000)	0.000(0.000,1.000)	1.000(0.000,3.000)	Z=-1.144	0.23323	0.63891
**lymphatic.metastasis**				χ2 = 1.350	0.24528	0.63891
no	15 (50.0%)	7 (70.0%)	8 (40.0%)			
yes	15 (50.0%)	3 (30.0%)	12 (60.0%)			
**stage**				χ2 = 2.769	0.42859	0.64803
I	1 (3.3%)	1 (10.0%)	0 (0%)			
II	13 (43.3%)	5 (50.0%)	8 (40.0%)			
III	13 (43.3%)	3 (30.0%)	10 (50.0%)			
IV	3 (10.0%)	1 (10.0%)	2 (10.0%)			
**Distant metastasis**				χ2 = 0.000	1.00000	1.00000
no	27 (90.0%)	9 (90.0%)	18 (90.0%)			
yes	3 (10.0%)	1 (10.0%)	2 (10.0%)			
**CEA.(ng/mL) (Median[Q1,Q3])**	4.080(1.987,9.543)	4.510(2.573,9.433)	4.080(1.762,9.762)	Z=0.748	0.46780	0.64803
**CA199.(U/ml) (Median[Q1,Q3])**	13.250(6.925,28.375)	12.350(7.725,23.025)	14.050(6.995,39.125)	Z=-0.682	0.50917	0.64803

Notest, t-test; Z, Mann-Whitney test; χ^2^, Chi-square test; SD, standard deviation; Q1, 1st Quartile; Q3, 3st Quartile.

At the species level, *Escherichia coli* was positively correlated with the number of lymph node metastases with a statistically significant difference (*p* < 0.05). *Parvimonas micra*, *Peptostreptococcus stomatis*, *Fusobacterium nucleatum*, and *Gemella morbillorum* were negatively correlated with CEA and the number of tumor lymph node metastases, with a statistically significant difference (*p* < 0.05). *Pantoea agglomerans* was negatively correlated with tumor size (**Figure 10B**).

## 4 Discussion

### 4.1 Association of flora diversity with CRC

The present study found that CRC patients had lower alpha diversity than healthy individuals, a result that is consistent with several studies (Zhao et al., 2024; Darnindro et al., 2025). A decline in alpha diversity may be attributed to the proliferation of pathogenic bacteria and a reduction in beneficial bacteria. In the gut of CRC patients, the enrichment of opportunistic pathogens (such as *Fusobacterium nucleatum* and *Escherichia coli*) and the depletion of butyrate-producing bacteria (such as *Roseburia* spp. and *Faecalibacterium* spp.) may impair intestinal barrier function and promote inflammation. This may lead to structural imbalance of the microbiota and impaired metabolic function, ultimately resulting in microbial dysbiosis (Li et al., 2023). The alpha diversity of tumor tissues was lower than that of adjacent tissues (**Figure 1E**), suggesting that metabolic characteristics of the TME (such as hypoxia and lactate accumulation) may directly inhibit microbial survival. For instance, elevated lactate levels within tumor tissues can alter the local pH, suppressing the growth of strict anaerobes such as *Bifidobacterium* spp., while simultaneously promoting the proliferation of acid-tolerant bacteria such as *Enterococcus* spp., thereby fostering a tumor-promoting microenvironment (Certo et al., 2021). Furthermore, fecal samples from CRC patients exhibited a reduced Shannon index (**Figure 1F**), indicating a trend toward simplified species distribution within their gut microbiota. This was characterized by the overgrowth of dominant taxa and a concomitant reduction in the abundance of commensal or beneficial bacteria, such as butyrate producers. Consequently, the ecological balance of the gut microbial community was disrupted.

Notably, the lower alpha diversity observed in cancerous tissues compared to adjacent paracancerous tissues (**Figure 1E**) reflects heterogeneity between the local microbial community within tumors and the overall gut microbiota. For instance, while the gut microbiota of CRC patients has shown enrichment of *Enterobacteriaceae*, the intratumoral communities are characterized by a predominance of *Fusobacterium nucleatum* (Obuya et al., 2022). This heterogeneity highlights the need for future studies to incorporate multi-compartment sampling (e.g., mucosal, tissue, and fecal specimens) to comprehensively assess microbiota alterations.

### 4.2 Alterations in microbial structure and its functional implications

The beta diversity analysis revealed a significant separation in the gut microbiota structure between CRC patients and healthy individuals (**Figures 3B, C**, p< 0.01). This finding has also been confirmed by other studies (Messaritakis et al., 2024). PCoA further demonstrated that although microbial composition at the genus level did not reach statistical significance between cancerous and adjacent paracancerous tissues (*p* = 0.326), a distinct separation trend was observed (**Figure 3A**). This trend may be potentially attributable to limited sample size or substantial inter-individual heterogeneity. Hierarchical clustering analysis (**Figure 2A**) revealed that cancerous tissue samples formed independent clusters, suggesting the presence of a tumor-specific microbial signature. This signature was characterized by an elevated abundance of *Escherichia-Shigella* and *Fusobacterium* (**Figure 6A**). Notably, *Escherichia-Shigella* contributes to CRC development through the production of the genotoxin colibactin, which induces DNA damage (Mo et al., 2020). *Fusobacterium* spp. promotes tumor proliferation by activating the Wnt/β-catenin signaling pathway (Valciukiene et al., 2023).

At the species level, CRC patients exhibited increased abundance of *Akkermansia muciniphila* (**Figure 9C**). Under healthy conditions, controlled mucin degradation by *Akkermansia muciniphila* promotes mucus layer renewal and provides nutrients (e.g., SCFAs) to other commensals, thereby supporting barrier function. However, recent studies have indicated that in advanced CRC stages, *Akkermansia muciniphila* may exacerbate tumor cell proliferation and invasion through excessive mucosal degradation. Furthermore, disruption of the mucus layer may activate the TLR4–NF-kB pathway, driving macrophage polarization toward the M1 phenotype and amplifying inflammatory responses within the TME (Zhang et al., 2024). This context-dependent functional duality underscores the stage-specific nature of microbial contributions to disease pathogenesis, mandating future analyses that stratify patients according to clinical disease stage.

### 4.3 Clinical utility of the GMHI and MDI

This study revealed that the GMHI was significantly higher in paracancerous tissues compared to cancerous tissues (*p* < 0.001), whereas the MDI showed an inverse pattern (**Figures 4A, E**). The reduced GMHI likely reflects depletion of protective taxa, such as *Blautia* spp. and *Faecalibacterium* spp., within the TME. This depletion may remodel the TME through multiple pathways, including metabolic dysregulation, immunosuppression, expansion of pro-tumorigenic bacteria, therapy resistance, and dysregulated cross-organ signaling. Notably, *Blautia* and *Faecalibacterium* are major producers of SCFAs, such as butyrate and propionate. SCFAs enhance antitumor immunity by inhibiting histone deacetylase (HDAC) activity and suppressing pro-inflammatory cytokine release (e.g., IL-6, TNF-α) via activation of G protein-coupled receptors (e.g., GPR43/109A). Specifically, butyrate inhibits tumor cell proliferation through HDAC suppression (Jiang et al., 2024). Conversely, elevated MDI correlated with enrichment of pathogenic bacteria, such as the pathobiont *Bacteroides fragilis*, which secrete enterotoxins that activate the STAT3 signaling pathway, thereby promoting CRC stem cell expansion (Zhou et al., 2023).

Healthy individuals exhibited significantly higher GMHI values than the CRC patients (**Figure 4B**), suggesting that GMHI may serve as a complementary biomarker for CRC screening. Furthermore, fecal samples demonstrated a higher GMHI than the cancerous tissue samples (**Figure 4C**), indicating that the stool microbiota retained residual healthy signatures while the local tumor-associated microbiota exhibited more severe dysbiosis. Our analysis revealed significant compositional differences between cancerous and adjacent paracancerous tissues in CRC patients, with adjacent tissues already displaying dysbiotic features resembling those in the cancerous tissues. This suggests that microbial imbalance may precede tumorigenesis and become progressively compartmentalized during cancer progression (Mo et al., 2020). As a distal sampling compartment, the fecal microbiota may retain stronger systemic signatures of host–microbiota interactions (e.g., immunomodulatory metabolites). The compositional divergence between fecal and tumor-resident microbiota reveals the multi-layered nature of host–microbial crosstalk. Fecal communities reflect systemic ecological equilibrium with tumor-localized microbiota actively shaping the microenvironment and driving malignant transformation (Valciukiene et al., 2023). Collectively, these findings necessitate future investigations integrating multi-compartment sampling (feces, tumor tissue, adjacent mucosa) with spatiotemporal dynamic analyses to delineate the pathological contributions of microbiota throughout carcinogenesis.

## 5 Mechanistic roles of key bacterial genera

### 5.1 Pro-tumorigenic mechanisms of pathobionts

*Escherichia coli*: Strains producing colibactin induce double-strand DNA breaks through DNA alkylation. This damage activates host DNA damage response pathways (e.g., p53 signaling), but persistent genomic insult leads to genomic instability, thereby driving tumorigenesis (de Souza et al., 2024). In this study, an elevated abundance of *Escherichia coli* was observed in the cancerous tissues of CRC patients (**Figure 6A**), suggesting its potential synergistic role in tumorigenesis through the driver-passenger model. Colibactin initiates tumorigenesis by inducing critical oncogenic mutations (e.g., Adenomatous Polyposis Coli (APC), Kirsten rats arcomaviral oncogene homolog(KRAS)). Other gut microbes or inflammatory microenvironments may further promote tumor progression. For instance, colibactin-induced cellular senescence is accompanied by a senescence-associated secretory phenotype (SASP), which recruits immune cells and fosters a tumor-promoting microenvironment (Dalmasso et al., 2024).

*Bacteroides fragilis*: Enterotoxigenic *Bacteroides fragilis* (ETBF) was significantly enriched in both cancerous tissue samples and fecal samples of CRC patients. The *Bacteroides fragilis* toxin (BFT) gene carried by ETBF strains is recognized as a pivotal oncogenic driver. ETBF promotes carcinogenesis through a toxin–inflammation–genomic instability–metabolic dysregulation cascade, establishing a tumor-promoting microenvironment and serving as a key driver in CRC pathogenesis (Scott et al., 2022).

*Akkermansia muciniphila*: This bacterium exhibits a context-dependent role in CRC, functioning as a double-edged sword. It may prevent tumorigenesis by maintaining barrier integrity, yet contribute to tumor progression via mucin degradation in the TME. Through mucin degradation, *Akkermansia muciniphila* generates metabolites, including SCFAs, which promote mucus layer renewal and enhance tight junctions between intestinal epithelial cells, thereby reducing pathogen and toxin translocation. Additionally, its outer membrane proteins (e.g., Amuc_1100) activate Toll-like receptor (TLR) signaling pathways, inducing regulatory T cell (Treg) differentiation and mitigating chronic inflammation—a key contributor to CRC pathogenesis (Fan et al., 2023). A study (Zhu et al., 2023) have reported enrichment of *Akkermansia muciniphila* or its genetic signatures in tumor tissues of advanced-stage CRC patients. This enrichment may promote cancer cell proliferation by activating oncogenic pathways such as Wnt/β-catenin. Additionally, mucin degradation products (e.g., sialic acid) may be utilized by tumor cells to support immune evasion or metastatic progression.

### 5.2 Protective roles of commensal bacteria

*Bifidobacterium*: A higher abundance of *Bifidobacterium* was observed in healthy individuals (**Figure 9B**). These bacteria enhance intestinal barrier function by secreting exopolysaccharides (EPS) and suppressing excessive inflammatory responses by inducing regulatory T cell (Treg) differentiation (Kainulainen et al., 2022).

*Faecalibacterium prausnitzii*: A study (Nittayaboon et al., 2022) have documented significantly reduced abundance of *Faecalibacterium prausnitzii* in the gut of CRC patients. As a primary butyrate producer, its depletion (**Figure 6C**) likely contributes to diminished colonic butyrate concentrations in CRC. Butyrate exerts tumor-suppressive effects through HDAC inhibition, inducing cancer cell apoptosis, suppressing proliferation, and reversing epithelial-mesenchymal transition (EMT). The loss of butyrate-mediated HDAC inhibition may consequently promote tumor progression (Luo et al., 2023).

## 6 Microbiota heterogeneity

This study revealed distinct microbial compositions across tumor tissues, adjacent tissues, and fecal samples (**Figures 5**, **6**). For instance, the cancerous tissue samples exhibited a significantly higher abundance of *Methylobacterium* (**Figure 9D**). This enrichment may promote tumor progression through two putative mechanisms: exacerbating formaldehyde-induced DNA damage via local formaldehyde accumulation, and modulating the immunosuppressive microenvironment through pathways such as TGF-β signaling (Dai et al., 2024). Conversely, the enrichment of *Blautia* spp. in fecal samples from CRC patients (**Figure 6D**) may indicate an attempted host-mediated restoration of microbial homeostasis.

This heterogeneity underscores that a single sample type (e.g., feces) may inadequately capture the complexity of the TME’s microbiota. Integration of multi-omics data (such as metagenomics and transcriptomics) could enable more precise functional characterization of microbial communities. Furthermore, the observed correlation between tumor staging and microbial abundance—exemplified by the association of *Fusobacterium nucleatum* with advanced-stage CRC (Yu et al., 2025)—requires rigorous validation.

## 7 Association analyses between microbial dynamics and clinical parameters

This study revealed significant correlations between specific bacterial genera and clinicopathological features in CRC tissues. *Escherichia-Shigella* abundance was positively associated with tumor size, while *Methylobacterium/Micrococcus* levels showed a significant association with tumor stage.

*Escherichia-Shigella* Genus: This genus belongs to the family Enterobacteriaceae. The primary species includes *Escherichia coli*, the most prevalent member within this genus. It ubiquitously colonizes the intestinal tracts of humans and animals, and it functions as a commensal bacterium while also representing a significant opportunistic pathogen.

*Shigella* comprises four principal species: *Shigella dysenteriae*, *Shigella flexneri*, *Shigella boydii*, and *Shigella sonnei*.

This study identified a significant positive correlation between the abundance of *Escherichia-Shigella* in tumor tissues and tumor size (*p* < 0.05), consistent with multiple recent investigations. Research demonstrates that *Escherichia coli* strains harboring the polyketide synthase (pks) genomic island secrete the genotoxin colibactin, which induces double-strand DNA breaks in host cells. This can lead to chromosomal instability and carcinogenic mutations, which are closely associated with the occurrence of tumors. Critically, such DNA damage may accelerate tumor cell proliferation, leading to increased tumor volume. Notably, the enrichment of colibactin-producing *Escherichia coli* (CoPEC) in CRC tumor tissues shows a positive correlation with tumor burden (Dalmasso et al., 2024). Our findings support this conclusion, indicating that *Escherichia-Shigella* colonization is closely related to tumor growth: the over proliferation of *Escherichia-Shigella* frequently coincides with depletion of beneficial taxa (e.g., *Faecalibacterium*, *Bifidobacterium*), thereby disrupting microbial homeostasis. Specifically, the reduced abundance of *Bifidobacterium*, which exhibits documented antitumor activity, may compromise the endogenous suppression of tumor growth (Chen et al., 2025). The genus *Escherichia-Shigella* is significantly enriched in the intestines of patients with colorectal cancer (especially in the early stage), which is associated with the processes of inflammation promotion and carcinogenesis (Kim et al., 2025). In summary, the *Escherichia-Shigella* genus promotes colorectal tumor growth and malignant progression through multifaceted pathways, including genotoxicity, inflammation induction, microbial dysbiosis, and metabolic disruption. The observed positive correlation between its abundance and tumor size highlights its close association with the occurrence and progression of colorectal cancer. However, the precise molecular mechanisms warrant further experimental validation.

This study revealed a significant association between the abundance of *Methylobacterium/Micrococcus* and tumor stage, suggesting this genus may influence CRC progression by modulating the TME. Research has demonstrated that *Methylobacterium* downregulates TGF-β expression and reduces CD8+ tissue-resident memory T cells (TRM). Murine models of primary gastric cancer confirm that these alterations promote tumor development (Peng et al., 2022). As a putative TME modulator, *Methylobacterium* may influence CRC through analogous mechanisms, such as metabolite-mediated signaling or immunomodulation. However, validation of its distinct pathogenic pathways requires further investigation (Wang et al., 2022). Metabolites derived from *Methylobacterium* may influence tumor progression. Bacterial small molecules such as methylmalonic acid have been shown to promote cancer metastasis, as evidenced in lung cancer models, and may similarly feed into CRC pathogenic cascades (Tanaka et al., 2025). While direct evidence in CRC remains limited, these findings suggest that microbial metabolites may influence CRC staging and progression through analogous mechanisms. Studies have reported that elevated serum methylmalonic acid levels in elderly patients correlate with CRC advancement, potentially promoting tumor invasion via activation of the TGF-β/Smad signaling pathway (Hu et al., 2023).

*Parvimonas micra* demonstrates significantly elevated abundance in CRC patients compared to healthy controls. Its levels correlate with tumor grade and molecular subtypes, particularly the CMS1 subtype. *Parvimonas micra* promotes CRC initiation and progression through immune activation—evidenced by increased CD69+ T lymphocytes and HLA-DR+ B lymphocytes and intestinal microenvironment remodeling. Furthermore, this pathobiont upregulates the Ras/ERK/c-Fos signaling pathway to accelerate CRC advancement. *In vivo* models have confirmed that *Parvimonas micra* accelerates colon tumorigenesis and significantly increases tumor burden (Chang et al., 2023). Despite the established roles of *Parvimonas micra* in CRC, the current literature lacks explicit evidence linking its abundance directly to CEA levels or lymph node metastasis status.

A study (Plaza-Diaz et al., 2024) has reported significant enrichment of *Gemella* species in both saliva and tumor tissues from oral squamous cell carcinoma (OSCC) patients, suggesting a potential role in the TME. However, current evidence has not yet determined whether *Gemella* infection directly influences lymph node metastasis burden or underlying mechanisms. Thus, the relationship between *Gemella* colonization and tumor lymph node metastasis represents a critical avenue for further investigation.

Study (Tsoi et al., 2017) found that *Peptostreptococcus* is a key genus in the colorectal “adenoma–carcinoma” related bacterial network and can serve as a potential marker for the diagnosis and treatment of colorectal cancer (CRC) and precancerous lesions. Study has confirmed that the abundance of *Peptostreptococcus* increases significantly during the progression of the colorectal “adenoma–carcinoma” sequence (Tsoi et al., 2017). Peptostreptococcus can directly or indirectly promote tumor cell proliferation through bacterial surface proteins, thereby accelerating tumor progression. After antibiotic treatment to eliminate endogenous microorganisms in APCMin/+ mice, continuous oral gavage of *P. stomatis* or *P. anaerobius* significantly increased the incidence of high-grade dysplasia, adenomas, and adenocarcinomas in the colorectum (Long et al., 2019). However, current research on *Peptostreptococcus* remains limited to its unidirectional carcinogenic effects. Current evidence has not established direct relationships between *Peptostreptococcus* abundance and CEA levels or lymph node metastasis burden.

Although this study has revealed significant associations, the cross-sectional design precludes the determination of whether the observed microbial alterations represent causal drivers or consequences of CRC. Causal relationships warrant experimental validation through organoid co-culture models and gnotobiotic animal studies. Future studies could establish spatiotemporal dynamic models to explore whether it plays a bidirectional regulatory role in CRC progression and treatment outcomes.

## 8 Geographic and population-specific disparities

Distinct microbial signatures characterize CRC patients across geographic regions. For instance, Thai populations exhibit *Prevotella* depletion associated with CRC (Iadsee et al., 2023), whereas our Chinese cohort demonstrates *Bacteroides* enrichment as a defining feature (Wang et al., 2023) (**Figure 6B**). These variations are likely mediated by dietary patterns, such as high-fiber versus high-fat intake (Gamage et al., 2024), underscoring the potential for dietary interventions in CRC prevention.

## 9 Methodological constraints and research trajectories: delineating limitations and forging translational pathways

Pre-analytical factors: Stool sample collection, storage temperature, DNA extraction batch, and PCR cycle number varied among participants; these uncontrollable differences could introduce technical variability that biases the observed microbiome profiles. The study employed 16S rRNA amplicon sequencing, which only provides compositional information at the genus and species level and cannot measure gene content, gene expression, or metabolite levels; therefore, direct functional assessment of the implicated taxa is not possible.

While this study reveals microbiota–CRC associations, several limitations warrant acknowledgment. The modest sample size may constrain statistical power, and the cross-sectional design inherently precludes causal inference between microbial shifts and colorectal carcinogenesis. Furthermore, viral and fungal microbiota components remain uncharacterized. To address these gaps and advance the field, future research should integrate multi-omics technologies, animal models, and clinical trials to mechanistically dissect host–microbe interactions, concurrently developing novel therapies targeting gut and intratumoral microbiota. Critical priorities include implementing longitudinal cohorts to resolve microbial dynamics during tumor progression, combining metabolomic and immunomic profiling to decipher microbiota–host crosstalk, establishing multi-omics diagnostic frameworks, and engineering personalized therapeutic strategies against pathogenic consortia.

## 10 Conclusion

Colorectal cancer (CRC) patients frequently exhibit altered gut microbiota composition, characterized by reduced alpha diversity. Cancerous tissues often show lower microbial diversity compared to adjacent non-cancerous tissues, suggesting potential microbial dysbiosis in the tumor microenvironment. Studies have observed enrichment of specific bacterial taxa, including *Escherichia-Shigella,Peptostreptococcus* and *Fusobacterium*, in tumor-associated niches, while butyrate-producing bacteria such as *Blautia* and *Faecalibacterium* tend to be more abundant in healthy individuals and adjacent tissues.

The observed associations between microbial alterations and CRC have led to investigations of potential mechanisms. Some studies suggest that certain bacterial metabolites, including lactate and butyrate, may influence the tumor microenvironment, though the causal relationships require further validation. Regional differences in microbiota composition have been noted, with Chinese CRC patients showing distinct *Bacteroides* enrichment patterns compared to Western populations exhibiting *Prevotella* depletion.

Current research explores the potential of microbiota-based indices, including GMHI and MDI, as complementary screening tools. However, their clinical utility requires validation in large-scale prospective studies. While fecal microbiota signatures may reflect host-microbe interactions, their diagnostic accuracy and clinical applicability remain to be established.

The relationship between gut microbiota alterations and CRC represents an active area of investigation. Understanding these associations may contribute to the development of future diagnostic and therapeutic strategies, though current evidence supports correlation rather than causation, and clinical applications require rigorous experimental validation.

## Data Availability

The original contributions presented in the study are included in the article/supplementary material. Further inquiries can be directed to the corresponding author.
